# Relationship between Tasks Performed, Personality Traits, and Sleep Bruxism in Brazilian School Children - A Population-Based Cross-Sectional Study

**DOI:** 10.1371/journal.pone.0080075

**Published:** 2013-11-14

**Authors:** Junia Maria Serra-Negra, Saul Martins Paiva, Mauro Henrique Abreu, Carmen Elvira Flores-Mendoza, Isabela Almeida Pordeus

**Affiliations:** 1 Department of Pediatric Dentistry and Orthodontics, Universidade Federal de Minas Gerais, Belo Horizonte, Minas Gerais, Brazil; 2 Department of Community and Preventive Dentistry, Universidade Federal de Minas Gerais, Belo Horizonte, Minas Gerais, Brazil; 3 Department of Psychology, Universidade Federal de Minas Gerais, Belo Horizonte, Minas Gerais, Brazil; University of Rochester, United States of America

## Abstract

**Background:**

Tasks can be instruments of stress and may affect the health of children. Sleep bruxism is a multifactorial sleep-related movement disorder that affects children and adults. The aim of the present study was to analyze the association between children’s tasks, personality traits and sleep bruxism.

**Methods And Findings:**

A cross-sectional, population-based study of 652 randomly selected Brazilian schoolchildren (52% of whom were female), aged from 7 to 10 years was conducted in the city of Belo Horizonte, Brazil. A questionnaire based on criteria proposed by the American Academy of Sleep Medicine (AASM) was completed by parents. In addition, the Neuroticism and Responsibility sub-scales of the Big Five Questionnaire for Children (BFQ-C) were administered to the children. Psychological tests were administered and evaluated by psychologists. The Social Vulnerability Index from the city council database was used to determine the social classification of the families. Chi-square and Poisson regression statistical tests were used with a 95% confidence interval. The majority of families were classified as having low social vulnerability (61.3%), whereas, 38.7% were classified as having high social vulnerability. Regarding extracurricular activities, the majority of girls performed household work (56.4%) and some artistic activity (51.3%) while sporting activities were most common among boys (61%). The results of the Poisson regression model indicated that sleep bruxism was most prevalent in children who scored highly in the Neuroticism sub-scale, and who frequently performed household tasks.

**Conclusion:**

Children whose personality domain has a high level of Neuroticism and who perform household chores imposed by the family are more vulnerable to sleep bruxism.

## Introduction

It is not uncommon today for children to perform a number of tasks: domestic labor, sports, dance, music, computer classes or foreign language learning [1]. Family influences may stimulate the performance of these tasks, pressuring children to participate, or even demotivating them [1]. 

Children are expected to participate in family-care tasks [2-4]. The household tasks that children do are linked strongly to gender roles. Girls are asked to perform tasks inside the house, such as cleaning and cooking, while boys are expected to complete jobs outside the house, such as mowing the lawn and washing the car [5]. Girls devote more time to household labor than boys [4]. A study of schoolchildren in Ohio found that a higher percentage of boys participated in sporting activities, while the performing arts were a more common choice among girls [1].

Expectations of children's household work practices reflect other concepts, based on cultural values [3]. For example, cultures may differ in their expectations of when children should begin household work, and in the perceived value of children's work in the home [5]. Child labor is accepted in a number of cultures [3,6,7] and child slavery is even described in some societies [8]. 

For a child to be healthy, his or her emotional and physical health should be in harmony [9]. The family plays an important role in this harmony during infant development. The family influences the number and type of tasks performed by children. Anderson et al. [1] found that when children perceived that parents stimulated the performance of extracurricular tasks, they tended to be involved in a greater number of extra activities. Excessive involvement in tasks may lead the child to become overburdened. Overburdened children may become physically ill; therefore, there is a possible association between overburdening and disease [10].

Each child has different abilities. Not all children are capable of performing the same tasks [11]. Personality can also influence the performance of tasks [12]. Personality traits are related to individual ways of dealing with different situations [12-16]. Traits of neuroticism may result in reactions of anxiety and anger, whereas, responsibility/conscientiousness is expressed through self-discipline and a sense of duty [12-16]. Personality traits in childhood may also be observed in adulthood [17]. Depending on personality traits present during childhood, the individual may have difficulty dealing with pressure and conflict and subsequently suffer from stress [18]. Individuals who score highly for neuroticism tend to be emotionally hypersensitive, sensitive to ridicule, incapable of dealing with pressure, and panic easily in emergency situations [19,20]. Personality traits can also be associated with physical health such as nutritional disorders and sleep disturbance [18,19,21].

Bruxism is the habitual, involuntary grinding or clenching of the teeth [21-24]. This oral condition has a multifactor etiology that affects both children and adults [25-27]. Sleep bruxism is a type of sleep disorder [25,26]. Sleep plays an important role in children’s health [28,29]. The consequences of sleep bruxism include: muscle pain, headaches, tooth wear and tooth loss [27]. There are a number of theories that associate emotional factors to the triggering of sleep bruxism [14,18]. Individuals with stress and/or specific personality traits tend to release the tension accumulated during the day through sleep bruxism [14,18,21]. 

There is considerable discrepancy among the prevalence of bruxism (5.9% to 78%) [14,23-27]. A recent systematic review reported a range of 3.5% to 40.6% [28]. There are also divergences in data collection methods used to assess bruxism among children [26,30,31]. The difference in results among the various studies on bruxism in children demonstrates the need for further research on this subject [14,23,26,27,30]. An understanding of the factors that can initiate sleep bruxism is of paramount importance. Work on the prevention of damage caused by this condition at an early stage can lead to improvements in general health [21].

Studies of the association between children’s tasks and sleep bruxism were not found on the Pubmed and Scopus databases during a search performed in June 2013, using key-words: tasks, sleep bruxism and children. Therefore, it was considered important to study this association. Subsequently, the aim of the present study was to determine the association between children’s tasks, personality traits and sleep bruxism in Brazilian schoolchildren.

## Materials and Methods

### Ethics Statement

All participants signed a written consent form. The consent form model, as well as the study were approved by the Research Ethics Committee of the Federal University of Minas Gerais process number 032/05.

### Study Location and Design

A population-based representative study of the city of Belo Horizonte, Brazil was performed. Belo Horizonte is the state capital of Minas Gerais, located in south east Brazil. It has 2,238,526 inhabitants, with 182,891 children enrolled in the elementary school system [32]. Belo Horizonte has nine administrative districts. Sample distribution was proportional to the total population enrolled in schools for each of nine administrative districts of the city. One school was randomly selected in each district. After approval of the principals of the schools, 808 parents of children enrolled in the second grade were invited to take part in the study. The informed consent forms were sent with the children's homework. A total of 753 parents authorized the participation (93%). The children exhibited sufficient reading skills to understand the psychological tests and completely answered the two personality scales. However, a total of 101 children failed to fulfill this criterion and were excluded from the study (13.4%). The sufficient reading skill criterion was evaluated by the teachers of the schoolchildren. All children agreed to participate. There were no refusals. The final sample was composed of 652 children, aged from 7 to 10 years. 

### Sample Size Calculation

The estimation of the proportion equation allows one to calculate the sample size with a standard error of 5% or less, which is sufficient to reject the null hypothesis. A 99% confidence interval level and a 35% prevalence of sleep bruxism were used for the calculation. The prevalence of sleep bruxism was determined in a pilot study conducted with 175 schoolchildren between 7 and 11 years of age who were randomly selected from both private and state schools in Belo Horizonte; these children were not included in the study population of the present research. The minimum sample size to satisfy requirements was estimated at 603 children.

To ensure a proper sample representation, the sample distribution was determined in direct proportion to the distribution of the children according to city administrative district, institution type, and child’s age. To provide each member of the study population with an equal chance of inclusion, the choice of the institutions and children was randomized until the target number had been reached.

### Socioeconomic Classification

The Social Vulnerability Index (SVI) was employed to evaluate the socioeconomic profile of the sample [33]. This index measures social exclusion in the city of Belo Horizonte and encompasses over 20 variables that quantify access to housing, schooling, income, jobs, legal assistance, health, and nutrition. The SVI is classified in five categories. Class I represents regions with the greatest degree of vulnerability, while Class V represents those with the lowest degree of vulnerability. There is a strong association between the SVI of a school and the SVI of the residence of the student (rho = .78). For the statistical analysis, SVI was divided into two groups: “high” (classes I and II) and “low” (classes III, IV and V). 

### Instruments

#### Bruxism Questionnaire

A self-report questionnaire consisting of ten questions regarding children’s history of audible nocturnal teeth grinding, oral habits, medical history, children’s tasks and socio-demographic information, was sent to the parents [21,23]. Parents were instructed to observe and record, during a three-day period, the presence or absence of sleep bruxism with their children.

The diagnosis of sleep bruxism was determined using the classification criteria proposed by the American Academy of Sleep Medicine (AASM), namely: occurrence of audible nocturnal teeth grinding; no other medical or mental disorders (e.g., sleep-related epilepsy, accounts of abnormal movements during sleep); and no other sleep disorders (e.g., obstructive sleep apnoea syndrome) [34]. 

Regarding children’s tasks, three categories were described by parents: household work, sports and performing arts. Household work included making one’s own bed, sweeping and cleaning the house, and caring for younger brothers and sisters. Sporting activities included football, swimming, basketball, volleyball, judo and other martial arts; whereas, performing arts included ballet and other dance, musical instrument, theatre, drawing and/or painting, and sculpture classes. The presence, absence and type of children’s tasks were evaluated based on parents’ reports. The time spent on each activity was not recorded.

#### Personality Test

The BFQ-C personality test [12,15], based on the Five-Factor Model, which addresses psychological traits such as Neuroticism, Openness, Extraversion, Responsibility/Conscientiousness, and Agreeableness, was used [35]. Neuroticism and Responsibility/Conscientiousness were considered important as the former reflects vulnerability to negative emotions, anxiety, anger, guilt, and clinical depression, while the latter reflects self-discipline, behaving dutifully, and striving for achievement [35,36]. It is believed that higher scores regarding these traits may have an effect on the health of individuals, even young people [18,19,21]. The BFQ-C was originally developed and validated for use on Italian children [12]. Each scale includes 15 questions. The responses employ a five-point Likert scale ranging from “never” to “always.” The score ranges from 15 to 75 points for each scale. The Neuroticism and Responsibility scales have been translated and cross-culturally adapted to Brazilian Portuguese [20].

Before administration of the BFQ-C, a pilot study with 175 schoolchildren, ages ranging from 7 to 11 years, randomly selected from private and state schools, were performed. These children were not included in the main study. The result of the Intra-Class Correlation for test-retest reliability (interval of one month) was .80 for the Responsibility scale and .83 for the Neuroticism scale. The internal consistency index was within acceptable limits and demonstrated suitability for the age range of the present study. The BFQ-C was administered collectively in the classroom by two psychologists. The pilot study supported that changes to the methodology proposed were not required.

### Scoring System

For statistical purposes, the values of the Neuroticism and Responsibility scales were used. Median scores were determined for each scale and dichotomized into “high” and “low” levels [13]. Using the median of scores as a cut-off point has been used in some studies [12-14].

For the Neuroticism scale, scores equal to or higher than 37 points indicated “high neuroticism,” whereas, scores lower than 37 points indicated “low neuroticism.” For the Responsibility scale, scores equal to or above 54 points indicated “high responsibility” and scores below 54 points indicated “low responsibility.”

### Statistical Analysis

The data collected was digitized and organized into a database using the Statistical Package for the Social Sciences (SPSS) software program, version 21.0. The chi-square test was used to test associations between bruxism, children’s tasks and personality traits, with sleep bruxism being the dependent variable. The Poisson regression model with robust variance was used to determine adjusted prevalence ratios, and a 95% confidence interval was adopted. Poisson regression with robust variance is an option to estimate Prevalence Ratio in cross-sectionalo studies, when the dependent variable is binary [38,39].The variables included in the model were those considered of epidemiological importance and had a value of p <.25. The inclusion of variables was made one by one, where only those variable with a significance level of p<.05 were kept in the final model. The significance level was set at 5%.”

## Results

As can be seen in Table 1, the majority of parents/guardians reported that they were living together (67.0%), while 214 (33.0%) were separated. The majority of families were classified as having low social vulnerability (61.3%), whereas, 38.7% were classified as being of high social vulnerability.

**Table 1:** Association between children’s tasks and gender.

Gender	Households Tasks		PR (CI 95%)	p *
	No (%)	Yes (%)		
Male	264 (84.6)	48 (15.4)	0.77 (0.55-1.09)	.332
Female	278 (81.8)	62 (18.2)	1	
	Sports			
	No (%)	Yes (%)		
Male	226 (72.4)	86(27.6)	1.70 (1.32-2.94)	<.001
Female	285 (83.8)	55(16.2)	1	
	Art			
	No (%)	Yes (%)		
Male	305(97.8)	7(2.2)	0.36 (0.16-0.85)	.029
Female	321(94.4)	19(5.6)	1	

* Pearson Chi square test ;p = probability values;PR = prevalence ratio; CI = confidence interval; values in parentheses refer to the percentage between rows.

Regarding extracurricular activities, girls performed more household work (18.2%) than boys (15.4%). Girls performed more artistic activity (5.6%) compare to boys (2.2%) while sporting activities were most common among boys (27.6%) (see Table 1). 

Sleep bruxism was present in 230 children (35.3%). However, as the majority of children without bruxism (66%) pertained to the group exposed to lesser social vulnerability as compared to the 34% with bruxism, an association was not found between vulnerability and bruxism (p = .390) (see Table 2). Moreover, the majority of children without bruxism (67.3%) had parents living together (p = .048) (Table 2). There was no statistically significant difference between gender and sleep bruxism, despite a higher percentage among girls (38.2%; p = .099) (Table 2).

**Table 2:** Association between sociodemographic variables, children’s tasks, personality traits and sleep bruxism.

	Bruxism			
Variables	Absent (%)	Present (%)	PR (CI 95%)	p*
Gender				
Male	212 (67.9)	100 (32.1)	0.84 (0.68-1.03)	.099
Female	210 (61.8)	130 (38.2)	1	
Age				
< 8 years old	35 (60.3)	23 (39.7)	1.14 (0.81-1.59)	.464
>8 years old	387 (65.2)	207 (34.8)	1	
Social Vulnerability				.
Low	264 (66.0)	136 (34.0)	0.91 (0.74-1.12)	390
High	158 (62.7)	94 (37.3)	1	
Marital Status				
Live Together	296 (67.3)	144 (32.7)	1.24 (1.00-1.53)	048
Separated	124 (59.3)	85 (40.7)	1	
Households Tasks				
No	369 (68.1)	173 (31.9)	0.62 (0.50-0.77)	<.001
Yes	53 (48.2)	57 (51.8)	1	
Sports				
No	321 (62.8)	190 (37.2)	1.31 (0.99-1.74)	.052
Yes	101 (71.6)	40 (28.4)	1	
Art				
No	401 (64.1)	225 (35.9)	1.87 (0.84-4.14)	.081
Yes	21 (80.8)	05 (19.2)	1	
Neuroticism				
Low	203 (74.1)	71 (25.9)	0.62 (0.49-0.78)	<.001
High	219 (57.9)	159 (42.1)	1	
Responsibility				
Low	30 (78.9)	8 (21.1)	0.58 (0.31-1.00)	.059
High	392 (63.8)	222 (36.2)	1	

* Pearson Chi-square test; p = probability value; PR = prevalence ratio; CI = confidence interval; values ​​in parenthesis refer to the percentage between rows.

The evaluation of the ratio between the residual deviance of the model (459.614) and degrees of freedom (649) in Poisson regression has shown a value lower but around 1. It indicates that there was no overidispersion of our data. Besides, our model fits well, considering that the chi-square test of residual deviance had shown a p value equal to 1.0. We then developed a negative binominal model. The value of the ratio between the residual deviance of this model (374.597) and degrees of freedom (651) was lower than in the Poisson regression with robust variance [39].

Regarding children involved in household work, bruxism and personality traits, an association between high neuroticism (p<.001; PR 0.62; 95% CI 0.49,0.78) and household work (p<.001; PR1 0.62; 95% CI 0.50,0.77) was found. In addition, sleep bruxism was more prevalent for the group of children with a high level of neuroticism (PR = 1.57; 95% CI 1.25,1.98) as well as for the group of children who performed household work (PR = 1.55; 95% CI 1.25,1.91) (see Table 3).

**Table 3:** Poisson regression model regarding household works, neuroticism level associated to sleep bruxism among schoolchildren.

Variable	PR (CI 95%)	p
Household tasks		
No	1	
Yes	1.55 (1.25-1.91)	<.001
Neuroticism		
Low	1	
High	1.57 (1.25-1.98)	<.001

PR = prevalence ratio; p = probability value

## Discussion

The present study evaluated the association between sleep bruxism, personality and tasks performed by children. To test the association, parents were requested to complete a questionnaire regarding prevalence of bruxism, characteristics of their children, and the types of tasks that they performed at home. The personality traits of children were also measured. Several points of interest were found. 

First, no significant difference was found between genders regarding sleep bruxism. This result is in contrast to the study conducted by Lam, Zhang, Li and Wing [26] who found a greater prevalence of sleep bruxism among boys. However, differences were found between genders regarding type of tasks performed. The majority of girls performed household work and arts, which is similar to the findings found by Arends-Tóth and Van de Vijver [5], while sporting activities were more common among boys. 

Secondly, household work is still identified as a largely female activity. The present study found that Brazilian families continue to sustain the culture of a division of tasks among their members. This tendency of family behavior has been noted in other studies [3,4,10]. The participation of the child in domestic tasks is seen as part of the educational process and stimulates discipline in childhood [10]. However, the child may be under stress if there is strong familial pressure involved in the performance of his or her tasks [1]. It is known that high stress levels are associated with sleep bruxism [18,29]. Thus, it is not surprising to find in the present study that the majority of children who performed work at home were those who suffered from sleep bruxism.

Thirdly, it was found that the majority of children without sleep bruxism had parents who lived together. Whether or not harmony at home affects the emotions of children is still a controversial subject [2,4,40]. 

Fourthly, children with high Neuroticism scores were more prone to sleep bruxism. If Neuroticism is characterized by the susceptibility to negative emotions which affect the adaptive functioning of an individual [19,20,37], household tasks could increase irritability among children with high Neuroticism [3,4]. On the other hand, pleasurable activities would have a positive influence on the quality of sleep [29,41,42]. Coincidently, in the present study, the majority of children who played some kind of sport did not suffer from sleep bruxism. This pleasurable sensation may be a protective factor for diminishing high levels of Neuroticism and sleep bruxism. 

Despite these findings, some limitations of the present study should be stated. For instance, the survey of types of tasks performed by children was based on information provided by parents. As a result, the non-evaluation of information from the children themselves about the type and quantity of work, and the feelings of pleasure or displeasure in performing the tasks, was a limitation of the present study. However, it is worth noting that studies regarding sleep bruxism and children’s tasks were not found in the Pubmed and Scopus databases in a search performed in June 2013. Thus, further investigation is necessary, especially in developing countries, in which household work performed by children is a common social phenomenon. 

## Conclusion

Based on the results of the present study, the following conclusions can be made:

1 Family characteristics and social behaviour can affect the emotional development of children.
2 Emotional aspects must be considered when investigating the presence of sleep bruxism in children.
3 Personality traits and children’s tasks are important factors that influence sleep bruxism among children.
4 The results of this investigation should advise health professionals of the importance of detailed interviews with families.
